# Old Materials for New Functions: Recent Progress on Metal Cyanide Based Porous Materials

**DOI:** 10.1002/advs.202104234

**Published:** 2021-11-26

**Authors:** Yi Xie, Rui‐Biao Lin, Banglin Chen

**Affiliations:** ^1^ Department of Chemistry University of Texas at San Antonio One UTSA Circle San Antonio TX 78249‐0698 USA; ^2^ MOE Key Laboratory of Bioinorganic and Synthetic Chemistry School of Chemistry Sun Yat‐Sen University Guangzhou 510006 China

**Keywords:** functionality, metal cyanide, pore chemistry, porous materials, Prussian blue

## Abstract

Cyanide is the simplest ligand with strong basicity to construct open frameworks including some of the oldest compounds reported in the history of coordination chemistry. Cyanide can form numerous cyanometallates with different transition metal ions showing diverse geometries. Rational design of robust extended networks is enabled by the strong bonding nature and high directionality of cyanide ligand. By virtue of a combination of cyanometallates and/or organic linkers, multifunctional framework materials can be targeted and readily synthesized for various applications, ranging from molecular adsorptions/separations to energy conversion and storage, and spin‐crossover materials. External guest‐ and stimuli‐responsive behaviors in cyanide‐based materials are also highlighted for the development of the next‐generation smart materials. In this review, an overview of the recent progress of cyanide‐based multifunctional materials is presented to demonstrate the great potential of cyanide ligands in the development of modern coordination chemistry and material science.

## Graphical abstract

Cyanide can be coordinated with different transition‐metal ions to yield a diverse class of cyanometallates featuring various geometries, which serve as building units enabling rational design of extended networks. New multifunctional porous materials are emerging thanks to the unique cyanide chemistry and the versatile combination of cyanometallates and organic linkers.

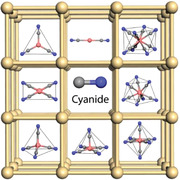

## 1 Introduction

The coordination chemistry of cyanide, the simplest ligand, can be dated back to the accidental discovery of Prussian blue in 1704, a widely used pigment. Since then, a wide range of transition metal cyanide complexes have been successfully synthesized and isolated with high diversity of bonding and structural features.^[^ ^1^ ^]^ The bonding nature of cyanide ligand with metals have been well established as *σ*‐donor and *π*‐acceptor.^[^ ^2^ ^]^ Metals of either high oxidation states or low oxidation states, such as Mo^V^, Mo^IV^, W^V^_,_ and W^IV^ in [M(CN)_8_]*^n^* ^−^ and Ni^0^ in [Ni^0^(CN)_4_], can be stabilized by cyanide of strong bonding characteristics.^[^ ^3^ ^]^ Such bonding nature and predictable directionality had attracted numerous interests for decades and facilitated the employment of cyanide ligand as bridging linker to construct coordination compounds from 0D clusters, 1D chains, 2D layers to 3D coordination networks. Thereby, the advances of cyanide chemistry had witnessed the breakthrough of coordination chemistry in many aspects, such as structural characterization, spectroscopic studies, and discovery of potential applications.

**Figure 1:**
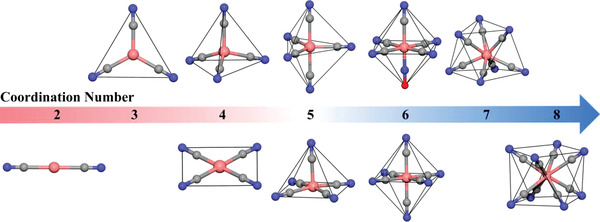
Geometries of common cyanometallate ions [M(CN)*_x_*]*^n^* ^−^ (*x* = 2 to 8). Pink, gray, and blue represent metal, carbon, and nitrogen atoms, respectively.

Most transition metals other than lanthanide are found to be capable of forming cyanide complexes, also referred as cyanometallates, whose geometries and symmetries, depending on the electronic configuration of center metal ions, varies from linear [M(CN)_2_]^−^ (M = Au, Ag and Hg), triangular [M(CN)_3_]^2−^ (M = Cu) and tetrahedral [M(CN)_4_]^2−^ (M = Zn, Cd, etc.) in d^10^ system, square planar [M(CN)_4_]^2−^ (M = Ni, Pt and Pd) in d^8^ systems, octahedral [M(CN)_6_]*^n^* ^−^ (*n* = 2 or 3, M = Fe, Co, Cr, etc.) in d^3‐6^ system, to dodecahedral [M(CN)_8_]*^n^* ^−^ (*n* = 3 or 4, M = Mo, W, Re, etc.) in d^1‐2^ system^[^ ^2^, ^4^ ^]^ (**Figure** **1**). What is more, partial replacement by other monodentate ligands, such as OH^−^, NH_3_, CO, NO^+^, SCN^−^, or ancillary ligands, such as Tp (hydrotris(pyrazolyl)borate), gives rise to a greater diversity of connectivity and functionalization with intrinsically responsive or catalytic properties in the coordination framework, as exemplified by nitroprusside‐based materials.^[^ ^5^ ^]^ However, the liability and low solubility of many reported cyanometallates are the major hindrance of their applications for extended coordination networks. In this context, stable cyanometallates, such as [M(CN)_2_]^−^, [M(CN)_4_]*^n^* ^‐^, [Fe(CN)_5_(NO)]^2‐^, [M(CN)_6_]*^n^* ^‐^ and [M(CN)_8_]*^n^* ^−^ have been well demonstrated to serve as linkers for multifunctional solid‐state materials.^[^ ^6^ ^]^ In particular, the family of Prussian blue analogs (PBA) is an outstanding example of porous materials composed of solely cyanometallates and transition metals, which have been extensively investigated in various fields due to the feasible modulation of chemical compositions and morphology as well as vacancy engineering.^[^ ^7^ ^]^ However, precise structural determination of PBA had long been a challenge and the vacancies were believed to be randomly distributed because the complexity of their crystal structures was somewhat hidden in the conventional X‐ray and neutron powder diffractions until very recently the arrangement of vacancies in Prussian blue was unraveled by X‐ray diffuse scattering patterns of single crystals.^[^ ^8^ ^]^ What is more, since the discovery of Hofmann clathrate [Ni(CN)_2_(NH_3_)]·C_6_H_6_,^[^ ^9^ ^]^ the incorporation of organic molecules as coligands expands the structural diversity of cyanide‐bridged networks even further to a stage where periodic coordination networks can be rationally designed and synthesized with suitable pore space, as evidenced by Hofmann‐type MOFs.^[^ ^10^ ^]^ Herein, cyanometallates, a large and miscellaneous class of old molecular building blocks, showcase its high potential in development of advanced multifunctional materials.

The variety of coordinated linkers in porous coordination polymers (PCP)^[^ ^11^ ^]^ or metal‐organic frameworks (MOF)^[^ ^12^ ^]^ is extensive, ranging from carboxylates to azolates,^[^ ^13^ ^]^ peptide,^[^ ^14^ ^]^ macrocyclic molecules,^[^ ^15^ ^]^ and even protein.^[^ ^16^ ^]^ Compared to other organic linkers, cyanide is one of the most easily available and inexpensive ligands but features diverse connectivity depending on the coordination with different metals. From a perspective of structural and topological design of extended networks, the linker connectivity can be systematically altered from low to high thanks to the variety of coordination numbers in the family of metal cyanide compounds, which gives rise to feasible tailoring of network dimensionality. Tremendous unique physical properties originated from the linkage nature of cyanide. For example, profound negative thermal expansion (NTE) behaviors have been discovered in some cyanide‐bridged compounds, which are typically originated from the transverse vibration/displacement of the diatomic ligands and metal ions.^[^ ^17^ ^]^ Notably, electronic/magnetic coupling enabled by the short linkage of cyanide is well‐established in many mixed‐valence and/or mixed‐metal compounds with efficient metal‐to‐metal charge transfer (MMCT) and superexchange pathways between metal ions.^[^ ^18^ ^]^ What is more, an increasing amount of functionalities have been witnessed in cyanide‐based porous materials, especially Prussian blue analogs, thanks to the fact that empty pore space, created by the M−CN−M' linkage, not only facilitates the mass transport for catalysis and ion conductivity/storage, but also promotes the investigation of gas separation/storage.^[^ ^19^ ^]^ More importantly, cyanometallates can also be considered as a class of functionalized ligands, as exemplified by linear and square planar [M(CN)*_x_*]*^n^* ^‐^ (*x* = 2, 4) featuring potentially accessible metal sites, the family of nitroprusside ions with noncoordinative and photoactive nitrosyl group and [M(CN)_8_]*^n^* ^‐^ with possibly uncoordinated CN groups exposed in the pore environment. Therefore, novel multifunctional materials can be judiciously targeted by taking the advantage of the versatile combination of cyanometallates and organic molecules.

In this review, we present the latest progress in cyanide‐based materials involving representative applications in the past decade, including gas adsorptions and separations, energy conversion and storage as well as spin‐crossover materials. More specifically, the structure‐property relationships will be discussed with the aim of illustrating the rich and unique coordination chemistry of cyanide‐based extended networks. In addition, the prospect and challenge of cyanide‐based materials as multifunctional materials in those fields will also be discussed.

## 2 Applications of Cyanide‐Based Porous Materials

### 2.1 Molecular Adsorption and Separation

The intrinsic empty space within cyanide‐based porous materials had motivated extensive efforts in exploring their applications in gas storage, such as hydrogen and carbon dioxide, in early stage.^[^ ^19^, ^20^ ^]^ The embedded pore space is accessible to external guest molecules and allows reversible uptake without sacrificing the structural integrity. Therefore, these materials can serve as porous media to capture gases and address the challenges in adsorptive molecular separations.

#### 2.1.1 C_2_H_2_ Purification

**Figure 2:**
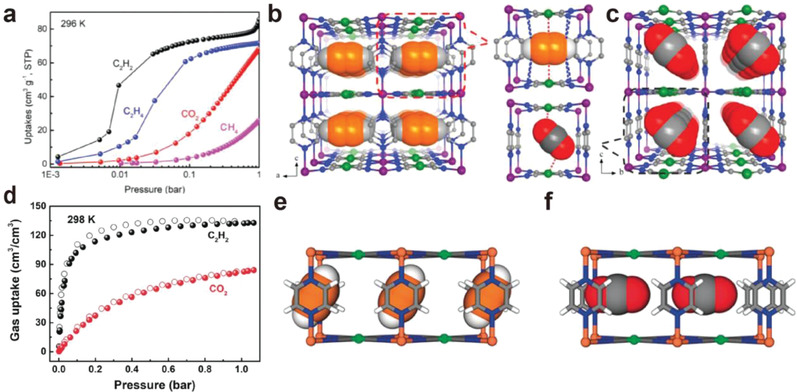
a) Single‐component adsorption isotherms of C_2_H_2_, C_2_H_4_, CO_2,_ and CH_4_ for ZJU‐74a at 298 K. The preferential binding sites for b) C_2_H_2_ and c) CO_2_ molecule in ZJU‐74a. Reproduced with permission.^[^ ^22^ ^]^ Copyright 2020, Wiley‐VCH. d) Sorption isotherms of C_2_H_2_ and CO_2_ for FeNi‐M′MOF. Neutron diffraction crystal structure of e) FeNi‐M′MOF⊃C_2_D_2_ and f) FeNi‐M′MOF⊃CO_2_. Reproduced with permission.^[^ ^23^ ^]^ Copyright 2020, Wiley‐VCH.

Acetylene is one of the most important chemical feedstocks in the petrochemical industry.^[^ ^21^ ^]^ By fine‐tuning the pore environment in the cyanide‐based materials, such as incorporating cyanometallate ligands with open metal sites (OMS) in the confined pore space, C_2_H_2_ can be selectively captured in the adsorbents with high selectivity and uptake. Qian et al. examined the separation performance of light hydrocarbons and carbon dioxide in a Hofmann‐type MOF, Co(pyz)[Ni(CN)_4_] (pyz = pyrazine), termed as ZJU‐74a.^[^ ^22^ ^]^ Record C_2_H_2_ adsorption capacity at low pressure (49 cm^3^ g^−1^ at 0.01 bar) and high selectivity for C_2_H_2_/CO_2_ (36.5), C_2_H_2_/C_2_H_4_ (24.2), and C_2_H_2_/CH_4_ (1312.9) were achieved under ambient conditions (**Figure** **2**a). Computational calculations revealed that the ultrahigh C_2_H_2_ capture capacity and selectivities were attributed to the sandwich‐like binding sites formed by adjacent open nickel sites from [Ni(CN)_4_]^2‐^ ligands, which provided strong interactions with the unsaturated bond in C_2_H_2_ molecules, while the interactions with CO_2_ molecules were weaker due to the failure to interact with two nickel sites in end on model in the confined pore space (Figure 2b,c).

The isoreticular MOF with the same [Ni(CN)_4_]^2‐^ ligand, Fe(pyz)[Ni(CN)_4_] (FeNi‐M'MOF) was investigated by Chen et al. and high volumetric C_2_H_2_ adsorption capacity (133 cm^2^ cm^−2^) with high C_2_H_2_/CO_2_ selectivity (24) was found in this material^[^ ^23^ ^]^ (Figure 2d). A high C_2_H_2_ productivity of 4.54 mmol L^−1^ was confirmed by column breakthrough experiments to evaluate practical separation performance. The preferential binding sites of C_2_H_2_ was identified by neutron powder diffraction studies, which revealed that the C_2_D_2_ molecules were located between the two pyz rings through *π*–*π* stacking (3.552 Å), while CO_2_ molecules were located between two nickel sites with weaker interactions (Figure 2e,f). Interestingly, substitution of the accessible metal site with Pt moiety was reported to negatively affected the C_2_H_2_/CO_2_ separation performance. Such adsorption behavior was ascribed to the stabilization on CO_2_ molecules by Pt moiety via Pt···C electrostatic interactions, as shown in a theoretical study on CO_2_ adsorption in Fe(pyz)[Pt(CN)_4_] by Sakaki et al.^[^ ^24^ ^]^ Therefore, the versatile tunability in these Hofmann‐type MOFs can be utilized for fine‐tuning the pore chemistry for optimal separation performance.

**Figure 3:**
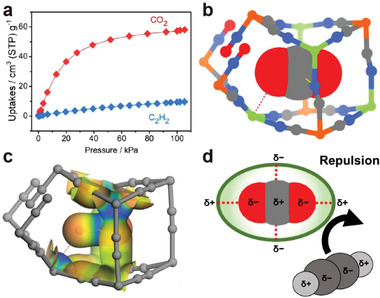
a) Single‐component adsorption isotherms of C_2_H_2_ and CO_2_ for Cd‐NP. b) Neutron diffraction crystal structure of Cd‐NP⊃CO_2._ c) Electrostatic potential (ESP) of Cd‐NP⊃CO_2_ mapped onto the electron density isosurface. d) Electrostatically driven CO_2_/C_2_H_2_ separation mechanism. Reproduced with permission.^[^ ^25^ ^]^ Copyright 2021, Wiley‐VCH.

Selectively removing CO_2_ impurity from crude C_2_H_2_ is a more feasible way to purify C_2_H_2_ because C_2_H_2_ with high purity can be directly produced in one single adsorption stage. However, it's still very challenging to rationally design an adsorbent to preferentially capture CO_2_ from C_2_H_2_ with high selectivity. Chen et al. reported a nitroprusside‐based ultramicroporous material Cd‐NP, Cd[Fe(CN)_5_(NO)], featuring compact pore space with complementary electrostatic potential for highly specific molecular recognition of CO_2_ over C_2_H_2_.^[^ ^25^ ^]^ Single component sorption isotherms showed a much higher uptake of CO_2_ (58.0 cm^3^ g^−1^) than that of C_2_H_2_ under ambient condition, affording a remarkable uptake ratio (6) and IAST selectivity (85) (**Figure** **3**a). Neutron powder diffraction and computational simulations studies indicated that the CO_2_ molecules were located in the cavity with a head‐on orientation toward the Cd center, through multiple weak host–guest interactions: Cd^*δ*+^···O^*δ*−^(CO_2_) interactions and van der Waals (vdW) interactions with the two cyanide ligands symmetrically (Figure 3b). Such restricted orientations and complementary host‐guest interactions electrostatically prohibited the adsorption of C_2_H_2_. (Figure 3c,d) Column breakthrough experiments validated the direct purification of C_2_H_2_ from CO_2_/C_2_H_2_ mixture under practical conditions.

#### 2.1.2 Hydrocarbon Separations

The separation and purification of hydrocarbons are crucial processes in the petrochemical industry to produce pure raw materials for the manufacturing of many chemicals. The application of Prussian Blue analogs (PBA) in light paraffin and olefin separations was first reported by Santamaría‐González et al. by examining the propylene/propane separation performance in two PBAs with different topology and same [Co(CN)_6_]^3‐^ ligand, rhombohedral Zn_3_[Co(CN)_6_]_2_ and classic cubic Cd_3_[Co(CN)_6_]_2_.^[^ ^26^ ^]^ Both compounds exhibited preferential adsorption for C_3_H_6_ with adsorption capacities of around 2 and 3 molecules of C_3_H_6_ per cavity in Cd_3_[Co(CN)_6_]_2_ and Zn_3_[Co(CN)_6_]_2_, respectively. What is more, the C_3_H_6_/C_3_H_8_ mixture was successfully separated by a column packed with Cd_3_[Co(CN)_6_]_2_ or Zn_3_[Co(CN)_6_]_2_, while higher separation capability and selectivity were exhibited in Cd_3_[Co(CN)_6_]_2._

Separation of hydrocarbons in humid conditions is even more challenging due to the competitive adsorption of water. Trens et al. reported the efficient separation of a mixed hydrocarbon (*n*‐pentane, *n*‐hexane, cyclohexane, and cyclohexene) in a humid atmosphere using a series of Prussian blue analogs.^[^ ^27^ ^]^ Among them, Co[Co(CN)_6_]_0.66_·5.2H_2_O, featuring the highest surface area, showed stronger affinity toward those hydrocarbons than water, and realized four‐component chromatographic separation with remarkable stability under humid conditions (50% humidity at 523 K). Notably, the crystallinity and separation performance were maintained after 30 cycles. The great stability endows Prussian blue analogs with promising potential in the challenging hydrocarbon separations.

#### 2.1.3 Harmful Gases Removal

The emission of harmful gases, such as ammonia, NO*_x_* and SO*_x_*, into the atmosphere is a major concern in public health protection. Reducing the concentration of these gases in the air is an efficient way to decrease PM 2.5. It remains challenging to use adsorbents with great stability and high adsorption capacity to remove these harmful gases at low concentration. In 2010, Behrooz et al. evaluated the SO_2_, H_2_S, and NO adsorption performance in Prussian blue analogs M_3_[Co(CN)_6_]_2_ *n*H_2_O (M = Co, Zn).^[^ ^28^ ^]^ In particular, Co_3_[Co(CN)_6_]_2_ was capable of capturing SO_2_, H_2_S, and NO with high uptake under ambient condition (2.5, 2.7, and 1.2 mmol g^−1^, respectively), and around 10 and 4.5 wt% of SO_2_, H_2_S at 0.1 bar, respectively. More importantly, the CO_2_ adsorption capacity of Co_3_[Co(CN)_6_]_2_ was not affected after the exposure to H_2_S.

Chemisorption‐like mechanism can achieve strong binding affinity and high adsorption capacity at low pressure. Real et al. reported the reversible chemisorption of SO_2_ in the Hofmann‐type MOF Fe(pyz)[Pt(CN)_4_], which exhibited sharp SO_2_ uptake below 50 mbar and reached saturation uptake of 1.32 mol of SO_2_ per mol of Fe(pyz)[Pt(CN)_4_] (3.03 mmol g^−1^) at 258 K.^[^ ^29^ ^]^ Interestingly, around 0.5 mol mol^−1^ of SO_2_ were trapped in Fe(pyz)[Pt(CN)_4_] after evacuation under same condition, whereas obvious reduced amount of captured SO_2_ was observed in Pd and Ni analogs, which suggested the unique chemisorptive binding on Pt sites. Single crystal diffraction experiments and DFT calculations had confirmed the coordination of SO_2_ on Pt site on the [Pt(CN)_4_]^2‐^ ligand with a square pyramid geometry (Pt−S 2.585(4) Å). What is more, the critical temperatures of the Fe(II) spin transition were shifted by 8−12 K due to the stabilization from the coordination of SO_2_.

**Figure 4:**
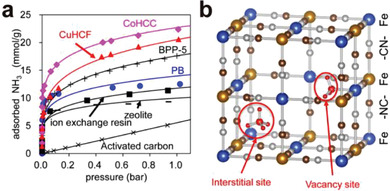
a) Comparison of NH_3_ adsorption isotherms of Prussian blue (PB), copper hexacyanoferrate (CuHCF), cobalt hexacyanocobaltate (CoHCC), other standard adsorbents. b) Crystal structure of Prussian blue and two adsorption sites. Reproduced with permission.^[^ ^30^ ^]^ Copyright 2016, American Chemical Society.

The adsorption capacity of ammonia in Prussian was extensively investigated by Kawamoto et al. In 2016, they evaluated the gaseous ammonia adsorption properties of two Prussian blue analogs, cobalt hexacyanocobaltate (CoHCC), Co[Co(CN)_6_]_0.60_, and copper hexacyanoferrate (CuHCF), Cu[Fe(CN)_6_]_0.50_.^[^ ^30^ ^]^ Prussian blue (PB) exhibited a high uptake of 12.5 mmol g^−1^ at 1 bar, exceeding those of most other standard NH_3_ adsorbents, while CoHCC and CuHCF, featuring higher surface area and density of vacancies, showed highest amount of adsorption capacity (21.9 and 20.6 mmol g^−1^) (**Figure** **4**a). IR spectra for NH_3_ loaded Prussian blue in ambient air revealed that two kinds of the adsorbed ammonia coexist at [Fe(CN)_6_] vacancy sites in Prussian blue: coordinative bounded with Fe sites and confined in the form of ammonia or NH_4_^+^ due to the protonation by the adsorbed water (Figure 4b). CoHCC can be regenerated in a vacuum and CuHCF can be recovered by acid washing. Furthermore, in 2018, the best‐performing material CoHCC was demonstrated to maintain high adsorption capacity at elevated temperature up to 250 °C, with a uptake of 25.2, 18.6, 8.6, and 2.1 mmol g^−1^ at 20, 100, 150, and 250 °C, respectively, out‐performing other standard porous materials.^[^ ^31^ ^]^ Fast adsorption‐desorption cycles were achieved even in the presence of water and oxygen molecules, which highlighted its feasibility of practical application via temperature swing adsorption (TSA).

Subsequently, the removal of trace ammonia is of great significance for air pollution control and many industrial productions, such as air treatment in semiconductors. Therefore, developing porous materials with capability of capturing ammonia at low concentration is crucial to the air purification industry. Kawamoto et al. examined the practical separations of trace ammonia (10 ppmv) from air in Prussian blue and its analogs via column breakthrough experiments.^[^ ^32^ ^]^ The PB and CoHCC removed over 99% of the ammonia from the air with an ammonia concentration of 10 ppmv with high capacities of 3.1 and 1.9 mmol g^−1^, respectively, higher than those of IE resins, zeolite 13×, and activated carbon. The PBAs can be recovered by pure water passing through the column, where ammonia was extracted from the column. The good recyclability of CoHCC was demonstrated by multiple adsorption/desorption tests with 10 ppmv of ammonia in air, in which it exhibited adsorption capacity of 1.1 mmol g^−1^ in 10 cycles. CoHCC was also capable of capturing ammonia released from livestock farms, which was saturated by water vapor. The concentration of ammonia was successfully reduced from 300 ppmv to 22 ppmv with a captured amount of 2.38 mmol g^−1^.

#### 2.1.4 Xylene Isomers Separation

**Figure 5:**
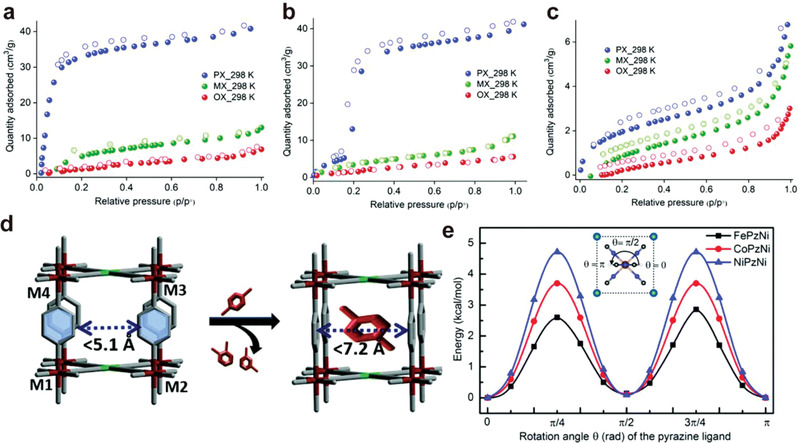
Pure component adsorption isotherms of xylene isomer in a) FePzNi, b) CoPzNi, c) NiPzNi measured at 298 K. d) Schematic representation of the pillar rotation induced by the adsorption of *p*X isomer. e) The potential energy surface for the rotation of the pyrazine pillars in three structures. Reproduced with permission.^[^ ^33^ ^]^ Copyright 2020, Royal Society of Chemistry.

The separation of xylene isomers (*para‐*, *meta‐*, and *ortho‐*xylene, abbreviated as *p*X, *m*X, and *o*X), important precursors for many chemicals, such as plastics, is highly challenging in the chemical industry due to their similar physiochemical properties. In 2020, Kitagawa et al. reported the adsorptive separation of *p*X from *o*X and *m*X in a series of isostructural Hofmann‐type porous coordination polymers (PCPs), M(Pz)[Ni(CN)_4_] with [Ni(CN)_4_]^2‐^ building blocks (M = Fe, Co, Ni, Pz = pyrazine).^[^ ^33^ ^]^ Vapor sorption isotherms showed high selectivity toward *p*X over *m*X and *o*X in FePzNi and CoPzNi: FePzNi exhibited a type‐I isotherm for *p*X with an uptake of 40 cm^3^ g^‐1^, in contrast to the much lower uptake of *m*X (10 cm^3^ g^−1^) and *o*X (5 cm^3^ g^−1^), while CoPzNi exhibited a gate‐opening effect for *p*X, followed by a uptake of 40 cm^3^ g^−1^ at *P*/*P*_o_ = 1 (**Figure** **5**a–c). Three isomers were adsorbed in NiPzNi with no noticeable difference. The vapor phase sorption of binary mixture in these materials have discovered that FePzNi and CoPzNi showed remarkable *p*X/*m*X and *p*X/*o*X selectivity and molecular sieving effect for *p*X/*m*X mixture in FePzNi. Computational studies were carried out to investigate the exceptional selectivity of FePzNi. The slightly longer interlayer distance (7.256(3) Å) induced a lower rotation barrier of Pz pillars, which allowed facile linker rotation to accommodate *p*X molecules with higher binding affinity, as determined by DFT‐D calculations (Figure 5d,e).

#### 2.1.5 Guest‐Induced Gate‐Opening Effect

Guest‐induced structural transformation of flexible coordination frameworks, also referred as gate‐opening effect, is a unique feature that had been intensively studied for various applications, such as proton conducting and molecular separation.^[^ ^34^ ^]^ Such gating effect triggered by guest molecule can be utilized for highly efficient molecular recognition.

In 2021, Ohba et al. reported an interesting guest‐selective gate‐opening effect in a new cationic 2D [Fe(CN)_6_]^2‐^‐based layers with a pseudo‐pillar ligand, 1,5‐anthraquinonedisulfonate (ADQS^2‐^).^[^ ^35^ ^]^ The grid‐like layers [Ni(dmen)_2_]_2_[Fe(CN)_6_] were supported by ADQS^2‐^ pillars (dmen = 1,1‐dimethylethylenediamine), residing at the middle of the layer grids via coulombic interactions, which afforded a pore system within the interlayer space. Two‐step and one‐step gate‐opening behaviors were observed for water and methanol in vapor sorption isotherms, respectively, while such phenomena were not triggered by larger molecules (ethanol) and gaseous molecules (N_2_ and CO_2_). The structural transformation was confirmed by PXRD, in which the interlayer spacing was significantly enlarged in the hydration process (11.08 Å), and the expansion was smaller upon sorption of methanol (10.89 Å). The small aperture in bottleneck‐type pore in dehydrated phase enabled size‐selective gate‐opening phenomenon that the diffusion of large molecules was prohibited.

**Figure 6:**
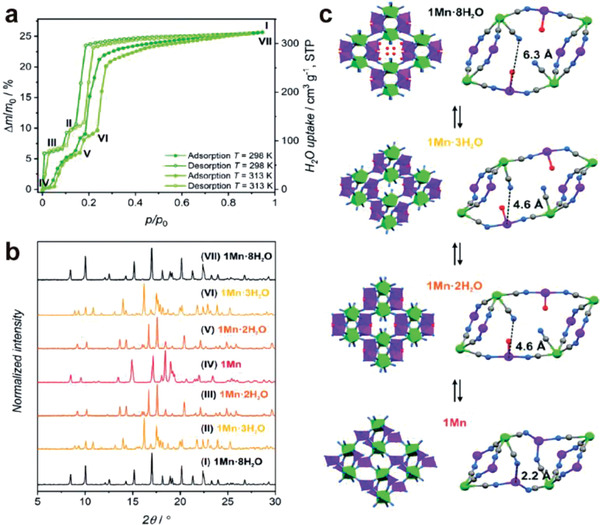
a) Water sorption/desorption isotherms for 1Mn·8H_2_O at 298 K and 313 K. b) PXRD patterns for different phases. c) Schematic representation of structural transformation of 1Mn·8H_2_O between different hydrated phases. Reproduced with permission.^[^ ^36^ ^]^ Copyright 2021, Royal Society of Chemistry.

Gate‐opening effect can even be driven by cleavage of the M–NC bond by guest molecules. Magott et al. reported stepwise water adsorption in {[Mn(imH)]_2_[Mo(CN)_8_]}*_n_* (1Mn; imH = imidazole) associated with large breathing effect.^[^ ^36^ ^]^ Three well‐defined steps in water adsorption were found, corresponding to three different phases as determined by powder X‐ray diffraction studies (**Figure** **6**a,b). Upon dehydration, four free water molecules and one coordinated water molecules in the hydrated {[Mn(imH)(H_2_O)_2_]_2_[Mo(CN)_8_]·4H_2_O}*_n_* (1Mn·8H_2_O) were removed in the first step affording 1Mn 3H_2_O, followed by further dehydration on another coordinated water molecules in the second step, giving 1Mn 2H_2_O (Figure 6c). More interestingly, since two cyanides in the [Mo(CN)_8_]^4−^ ligand were not coordinated to Mn in the hydrated phase, the full removal of coordinated water led to a new linkage of Mo–CN–Mn, forming a “crossed‐ladder” cyanide bridging pattern. Such phenomenon was found fully reversible with great stability in multiple dehydration–rehydration cycles. The selective gate‐opening effect in this nonporous 1Mn enabled high water uptake of over 25%, which demonstrated its potential for water harvest.

**Figure 7:**
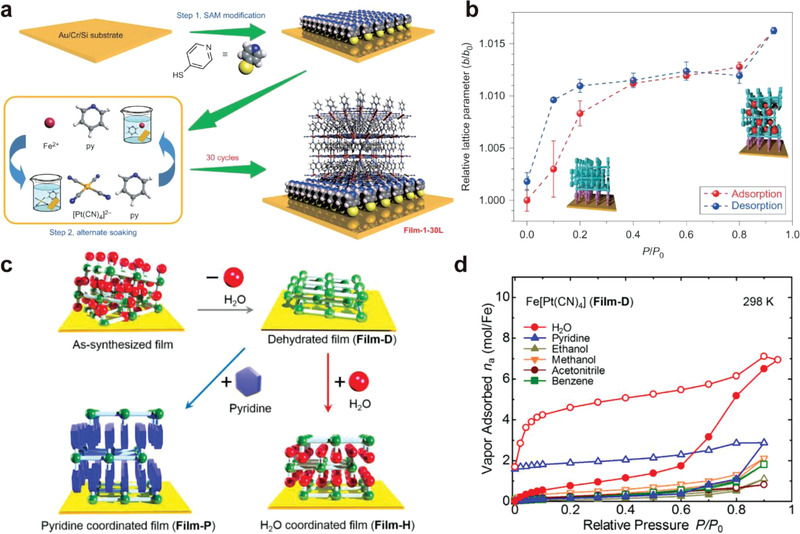
a) Schematic illustration of the thin‐film fabrication of Fe(py)_2_[Pt(CN)_4_] thin film via the LbL protocol. b) Relative lattice constant plotted against the relative pressure of ethanol vapor. Reproduced with permission.^[^ ^37^ ^]^ Copyright 2016, Nature Publishing Group. c) Two‐way structural transformation of dehydrated Fe[Pt(CN)_4_] thin film (Film‐D) to water/pyridine coordinated Fe(L)_2_[Pt(CN)_4_] film (L = water, pyridine). d) Vapor adsorption/desorption isotherms of Film‐D. Reproduced with permission.^[^ ^38^ ^]^ Copyright 2016, American Chemical Society.

However, the structural dynamic of flexible frameworks in reduced size or in thin film was rarely studied. In 2016, Kitagawa et al. reported the fabrication of the 2D Hofmann‐type MOF Fe(py)_2_[Pt(CN)_4_] (py = pyridine) thin film on Au/Cr/Si substrate through layer‐by‐layer (LbL) growth at the liquid/solid interface.^[^ ^37^ ^]^ The 2D layers consist of cyanide‐bridged Fe(II) ions and [Pt(CN)_4_]^2‐^ building blocks, and the pyridine molecules were coordinated to the Fe(II) ions forming *π*–*π* stacking with adjacent pyridine (**Figure** **7**a). Although this MOF shows no significant gate‐opening toward vapors of guest molecules in bulk due to the densely packed layers, when fabricated as thin film and downsized to16 nm thick, the resultant thin film, termed as film‐1‐30L, exhibited clear gate‐opening effect to many vapors, including ethanol, water, methanol, and acetonitrile. In situ powder XRD studies showed that the vertical interlayer spacing, represented by the 020 and 040 peaks, were expanded by 1.6% upon ethanol adsorption, allowing guest molecules to diffuse (Figure 7b). This work has demonstrated a strategy to manipulate the dynamic structural transformation of 2D cyanide‐based Hofmann‐type MOF by downsizing.

Such gate‐opening effect can be fine‐tuned by modifying the ligands coordinated on the Fe[M(CN)_4_] layers. The structural transformation is governed by the strength of interlayer interactions, dominantly from the *π*–*π* stacking of the coordinated ligand. In 2019, Martí‐Gastaldo et al. reported the fabrication of a series of Fe(II) Hofmann‐type MOF thin films (thickness < 30 nm) with different ligands (pyridine, pyrimidine, and isoquinoline).^[^ ^39^ ^]^ The stronger *π*–*π* stacking between isoquinoline (isoq) molecules in Fe(isoq)_2_[Pt(CN)_4_] films significantly suppressed the interlayer expansion upon solvent vapors. The maximum expansion was observed in the smallest methanol molecules (1.0%). The lattice expansion was weakened in bulkier solvent molecules, such as ethanol, acetonitrile, and toluene.

The pore space and the underlying host–guest chemistry can be optimized by incorporating hydrogen bonding between the ligands to modifying structural flexibility. In 2019, Real et al. reported the construction of isoreticular 2D Hofmann‐type MOF Fe(5‐NH_2_pym)_2_[M(CN)_4_] (M = Pt or Pd) with 5‐aminopyrimidine (5‐NH_2_pym) as ligands, in which the amine groups and aromatic nitrogen atoms served as hydrogen bonding donors and acceptors, enabling enough pore space for the accommodation of guest molecules.^[^ ^40^ ^]^ The as‐synthesized phase with water as guest molecules can be dehydrated and reversibly adsorb/desorb protic solvent molecules, such as water, methanol, and ethanol, as evidenced by single‐crystal diffraction studies. The vapor sorption isotherms of the bulk material showed gate‐opening effect for both compounds upon these solvent vapors, demonstrating the structural transformation that is not observed in the bulk prototypical Fe(py)_2_[M(CN)_4_] and its derivatives.

Interestingly, cyanide‐based 2D coordination network with no axial coordinated ligands were also reported to show gate‐opening effect toward guest molecules. In 2016, Kitagawa et al. reported chemisorption in a 2D‐layered MOF thin film, Fe[Pt(CN)_4_], driven by the coordination of guest molecules on the open metal sites.^[^ ^38^ ^]^ Synchrotron XRD studies revealed a AB packing model of the Fe[Pt(CN)_4_] layers in dehydrated Fe[Pt(CN)_4_] thin film (Film‐D), with great crystallinity (Figure 7c). Film‐D showed gate‐opening effect for water and pyridine molecules with a high uptake of around 7 and 2 mol mol^−1^ Fe in vapor sorption experiments, respectively. Around two molecules of water/pyridine per mol Fe remained in the desorption process, indicating a stoichiometric coordination to the open Fe sites (Figure 7d). The structural transformation of Film‐D to Film‐H and Film‐P upon water and pyridine vapors, respectively, were verified by the PXRD pattern with the analogs Fe(py)_2_[Pt(CN)_4_] and Fe(H_2_O)_2_[Ni(CN)_4_]·2(1,4‐dioxane). The crystal structure of Film‐D was completely transformed into Film‐H at *P*/*P*_0_ = 0.95 and the Film‐H phase was maintained after desorption, which suggested the irreversible and strong coordination of water on the layers.

### 2.2 Electrocatalysis

As the global economy grows rapidly, developing clean and sustainable energy sources to replace fossil fuels is in urgent need due to the limited energy supplies and the environmental pollution. The current energy crisis has threatened the human society and caused environmental problems.^[^ ^41^ ^]^ Hydrogen and fuel cells are the most promising technologies for clean energy to replace those traditional fossil‐based energy solutions due to the low environmental pollution and high energy conversion efficiency.^[^ ^42^ ^]^ Electrocatalytic water splitting is an efficient and practical strategy to produce hydrogen as a fuel, consisting of two important half‐reactions: hydrogen evolution reaction (HER) and oxygen evolution reaction (OER) which occur at the cathode and anode, respectively.^[^ ^43^ ^]^ Oxygen and hydrogen are generated from the oxidation of water or hydroxyl ions in OER and reduction of proton in HER, respectively. However, the development of these electrochemical reactions in clean energy industry suffered from the slow kinetics of these reactions and high overpotential to obtain desired currency density, which sets great challenge in design of new electrocatalyst for efficient and stable performance with low cost.^[^ ^44^ ^]^ Cyanide‐based materials, especially Prussian blue analogs, and its derivatives were extensively investigated as the new alternative catalysts in replacement of noble metal catalysts thanks to its facile and low‐cost synthesis and highly tunable features.^[^ ^45^ ^]^

The versatile compositions and functionalities in cyanide‐based materials, such as Prussian blue analogs, render them as new catalysts for water splitting. Galan‐Mascaros et al. reported the fabrication of Co‐Fe Prussian blue analogue thin films via chemical etching and its OER performance in neutral aqueous media.^[^ ^46^ ^]^ A small reversible Co^II^/Co^III^ redox couple at 0.89/0.98 V and a sharp onset at 1.15 V versus NHE (334 mV overpotential) were observed in cyclic voltammograms (CV) in pH 7 KPi + 1 m KNO_3_ electrolyte. CoFe showed great stability for keeping constant overpotential over 8 h at 1 mA cm^−2^ in the same electrolyte. The catalytic activity was found to be dependent on pH in the range of 2 to 13, whereas overpotential was significantly higher at pH = 1.

Similar to Prussian blue analogues (PBA), metal nitroprusside compounds and Hofmann‐type MOFs were also demonstrated as great candidates in water splitting. Wu et al. reported the fabrication of ultrathin CoFe‐nitroprusside, Co[Fe(CN)_5_(NO)]·4H_2_O, nanosheet on nickel foam, termed as CoFe‐PBA NS@NF, which showed ultralow overpotentials of 256 and 48 mV to drive 10 mA cm^−2^ for OER and HER in 1.0 m KOH solution, respectively. The alkaline electrolyzer can be driven at 10 mA cm^−2^ with only 1.545 V.^[^ ^47^ ^]^ Gao et al. examined the OER performance of a series of isoreticular Hofmann‐type MOFs, [MM']L[Ni(CN)_4_], (M and M' for Fe^2+^, Co^2+^, Ni^2+^; L for pyrazine).^[^ ^48^ ^]^ This series of materials served as a platform to investigate the OER catalytic activity based on different metal compositions and underlying modulation on electronic structure. Among them, the lowest overpotential of 300 mV at 10 mA cm^−2^ under pH = 13 was exhibited in CoFe‐PYZ ([FeCo]L[Ni(CN)_4_]) with a Tafel slope of 44 mV dec^−1^. DFT calculations revealed a reduced energy barrier was due to the synergistic effect between Fe^2+^ and Co^2+^.

**Figure 8:**
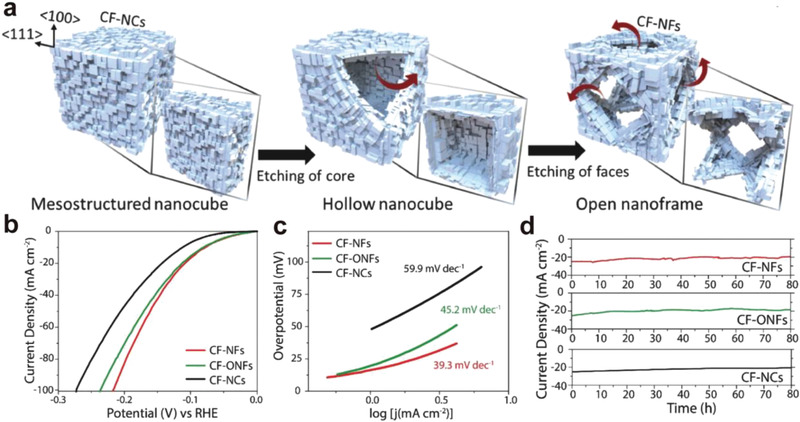
a) Schematic representation of the formation process of CF‐NFs from CF‐NCs. b) Polarization curves of CF‐NF, CF‐ONF, and CF‐NC measured in 1 m KOH. c) Tafel slopes for CF‐NF, CF‐ONF, and CF‐NC. d) Chronoamperometry tests for CF‐NF, CF‐ONF, and CF‐NC. Reproduced with permission.^[^ ^49^ ^]^ Copyright 2018, Wiley‐VCH.

The catalytic performance of the pristine cyanide‐based structures can be effectively tuned by engineering the morphology of nanocrystal, for example, control of excess defects and hollow structure. In 2018, Ho et al. reported the synthesis of CoFe‐PBA octopod nanoframes (CF‐ONFs) and nanoframes (CF‐NFs) from chemical etching of CoFe‐PBA nanocubes (CF‐NCs), with similar particle size of 550 nm^[^ ^49^ ^]^ (**Figure** **8**a). The face etching process yielded the hollow nanoframeworks of CF‐ONFs and CF‐NFs, while in CF‐ONFs, eight arms were preserved to extend toward the corners of the cube. The abundant surface defects and highly reactive high‐index facets significantly enhanced the catalytic performance of hydrogen evolution reaction (HER) in 1 m KOH. The overpotentials of CF‐NFs and CF‐ONFs for HER at a current density of 10 mA cm^−2^ are 78.1 and 82.6 mV, respectively (Figure 8b). These materials outperformed most other noble‐metal‐free HER catalysts under acidic and alkaline conditions. Tafel slopes of ≈ 39.3, 45.2, and 59.9 mV decade^−1^ for CF‐NCs, CF‐ONFs, and CF‐NCs confirmed that the HER kinetics was boosted within the hollow structures (Figure 8c). In addition, CF‐NCs, CF‐NFs, and CF‐ONFs showed exceptional stability under a currency density of −25 mA cm^−2^ for 80 h with little degradation of catalytic performance (Figure 8d).

Another strategy of increasing compositional heterogeneity and complexity was demonstrated by building heterogeneous hollow core–shell structure of Co–Fe PBA through one‐pot synthesis (reduction‐cation exchange strategy).^[^ ^50^ ^]^ The Fe^III^ in Co_3_[Fe^III^(CN)_6_]_2_ nanocubes were reduced by PVP to Fe^II^ species, which etched the face‐center positions and formed a shell of Fe^II^_3_[Fe^III^(CN)_6_]_2_ due to the lower *K*_sp_. Therefore, a complex nanoarchitecture (PBA‐5) composed of Fe‐rich shell and Co‐rich core was successfully synthesized, which enabled high surface area of 576.2 m^2^ g^−1^ and high OER catalytic performance. Remarkably low overpotential of 271 mV at 10 mA cm^−2^, Tafel slope (53.7 mV dec^−1^) and long‐term stability (24 h) were exhibited, highlighting the potential of compositional complexity in optimizing OER performance.

However, these cyanide‐based structures may act as precatalyst or precursor of high‐performance electrocatalysts. Zhang et al. reported the NiFe‐PBA (NF‐PBA) underwent complete activation of 10 h to an amorphous Ni(OH)_2_, which showed a remarkably low overpotential of 258 mV.^[^ ^51^ ^]^ The real active catalytic sites in the OER process were the nickel hydroxide, which was found to be deprotonated into NiOOH_2−_ *_x_* by Operando XAS. At high potential, Ni^4+^ ions were generated from the oxidation of Ni^2+^ ions to compensate the charge of proton and served as active sites enabling oxidized oxygen ions to act as electrophilic centers to facilitate the OER activity. What is more, transition metal hydroxide nanosheets can be derived by dispersing the 2D Hofmann‐type MOFs, M(H_2_O)_2_Ni(CN)_4_·4H_2_O (M = Mn, Fe, Co, and Ni), in aqueous KOH solution.^[^ ^52^ ^]^ PXRD and IR spectroscopy confirmed the complete and quick structural transformations. Among those resultant transition metal hydroxide nanosheets, Fe(OH)_3_‐NS exhibited the lowest overpotential of 271 mV at the currency density of 10 mA cm^−2^, comparable to the commercial IrO_2_, with a Tafel plot of 51 mV dec^−1^. The good catalytic performance in these derived materials and underlying structural transformations emphasize the importance of careful characterizations of real catalytically active sites.

In summary, the structural diversity and high tunability in composition and morphology of cyanide‐based materials facilitate the design of new derived materials for energy conversion. These structures provide a good platform to develop composite or derivatives materials for various energy conversion applications in the future.

### 2.3 Batteries

Advanced energy storage systems are in urgent need for the development of renewable energy sources. Batteries that possess high storage capacity, recharge efficiency, and long‐term stability are the cornerstone of energy storage devices. Among cyanide‐based materials, Prussian blue analogs have been widely explored in energy storage application for decades as the cathode/anode materials.^[^ ^53^ ^]^ The applications of Prussian blue analogs in this field benefited from several structural features: i) the charge‐carrier ions can be reversibly inserted in the open frameworks as counter‐ions; ii) potential scale‐up synthesis with low cost; iii) compositions of metal ions within the Prussian blue analogs and morphology can be rationally designed and tailored for on‐demand electrode synthesis and fabrication.^[^ ^54^ ^]^ In principle, the electrode capacity of this type of material relies on the redox reactions on the transition metal ions, which allows the insertion/extraction of the carrier ions. Therefore, precise control of electronic structure and defects are crucial to practical performance.

#### 2.3.1 Alkaline Ion (Li^+^/Na^+^/K^+^) Batteries

A series of PBA materials were evaluated as cathodes or anodes for alkaline ion (Li^+^/Na^+^/K^+^) batteries. Cui et al. reported the synthesis of CuHCF as cathode in K^+^‐ion batteries, which exhibited a specific capacity of 59.14 mAh g^−1^ at 0.946 V.^[^ ^55^ ^]^ Outstanding stability was demonstrated by 83% of retained capacity after 40 000 cycles at a 17 C rate. The feasibility of a series of Prussian blue analogs with different transition metal ions (Fe, Mn, Ni, Cu, Co, and Zn) as cathode materials in sodium ion batteries (organic liquid‐carbonate electrolyte) was systematically investigated by Goodenough et al. in 2012.^[^ ^56^ ^]^ KFe^II^Fe^III^(CN)_6_ exhibited a reversible capacity of ≈100 mAh g^−1^ and good reversibility in 30 cycles with a flat discharge capacity of over 70 mAh g^−1^. In 2014, two Prussian blue analogs, Fe_4_[Fe(CN)_6_]_3_ and FeFe(CN)_6_ were examined as cathode materials for Li‐ion batteries, which exhibited a charge capacity of 95 and 138 mAh g^−1^, respectively.^[^ ^57^ ^]^ FeFe(CN)_6_, with more Fe^III^ content, delivered 96 mAh g^−1^ after 80 cycles, demonstrating its good cyclability. However, the charge capacity and long‐term stability were still a challenge for this class of materials.

**Figure 9:**
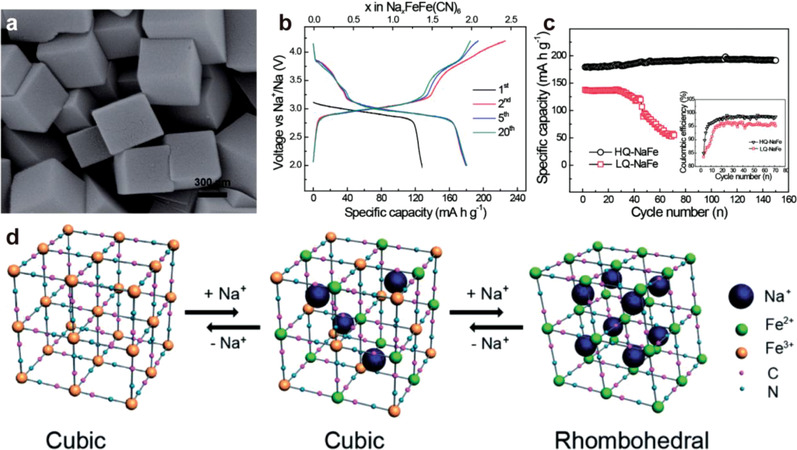
a) SEM image of HQ‐NaFe. b) Galvanostatic discharge/charge voltage profiles of HQ‐NaFe. c) Cycling performance of HQ‐NaFe and LQ‐NaFe under a current density of 25 mA g^−1^, with an inset showing Coulombic efficiencies of HQ‐NaFe and LQ‐NaFe. d) Schematic representation of the redox mechanism of HQ‐NaFe. Reproduced with permission.^[^ ^58^ ^]^ Copyright 2014, Royal Society of Chemistry.

Although the theoretical specific capacities of PBA materials with two redox‐active centers are predicted to be over 170 mAh g^−1^ for sodium ion batteries and 190 mAh g^−1^ for potassium ion batteries, structural defects and water content in as‐synthesized crystals significantly affect the ion storage and diffusion of ions.^[^ ^54^ ^]^ A facile synthetic method was reported to reduce zeolitic water content and [Fe(CN)_6_] vacancies through slowly growing high‐quality PB crystals Na_0.61_Fe[Fe(CN)_6_]_0.94_ (HQ‐NaFe)^[^ ^58^ ^]^ (Figure 8a). The sodium storage capacity in HQ‐NaFe was remarkably enhanced, reaching its theoretical value as the cathode for Na‐ion batteries (170 mAh g^−1^) with high cycling stability (no obvious capacity loss in 150 cycles), and high Coulombic efficiency (**Figure** **9**b,c). Interestingly, the structure of HQ‐NaFe was reversibly transformed into cubic NaFe^III^Fe^II^(CN)_6_ and rhombohedral NaFe^II^Fe^II^(CN)_6_ at low and high sodium loading in Na^+^ insertion/extraction process, respectively, which favored the accommodation of the sodium ions (Figure 9d). A similar defect engineering method was also proven effective in improving the Li‐ion storage performance of FeFe(CN)_6_ as cathode material.^[^ ^59^ ^]^ Such structural evolution in charge/discharge process is intriguing due to the different electrochemical properties exhibited in different phases. For instance, Goodenough et al. reported the formation of rhombohedral Na_2−*δ*_MnHCF induced by dehydration of interstitial water, which delivered 150 mAh g^−1^ with flat charge and discharge plateaus at 3.5 V and great rate capability and cycling performance (75% capacity retention after 500 cycles at 0.7 C).^[^ ^60^ ^]^

The PBA materials are also a class of promising electrode materials for aqueous electrolyte‐based K‐ion battery (KIB), which is a potential candidate for grid‐scale energy storage applications. Hu et al. reported an AKIB system fabricated with an Mn‐rich Prussian blue K*_x_* Fe*_y_* Mn_1_ *_−y_* [Fe(CN)_6_]*_w_* ·*z*H_2_O cathode, an organic anode (3,4,9,10‐perylenetetracarboxylic diimide) and a 22 m KCF_3_SO_3_ water‐in‐salt electrolyte.^[^ ^61^ ^]^ The PBA cathode exhibited a high capacity of 135 mAh g^−1^, a high capacity retention of 95% at 20 C and 70% at 100 C, and superior cycling stability (10 000 cycles). The overall AKIB system showed a high energy density of 80 Wh kg^−1^ with a high rate capacity of 20 C and wide range of temperature (−20–60 °C)

#### 2.3.2 Multivalent Ion Batteries

The abundant and inexpensive multivalent ions (divalent Mg^2+^, Ca^2+^, Zn^2+^ and trivalent Al^3+^) have attracted increasing interests because the energy density is much higher compared to monovalent ions from the doubly/triply charges per ion.^[^ ^62^ ^]^ It is estimated that the theoretical volumetric energy density of Mg (≈3833 mAh cm^−3^) is much higher than that of Li (≈2046 mAh cm^−3^). Therefore, multivalent ion batteries are promising as “beyond Li‐ion” technology for advanced energy storage solutions. However, the development of multivalent ion batteries is restricted by the low ion mobility resulted from their larger ion sizes and higher valences. Also, the stability and performance of candidate cathode materials are still underdeveloped. Prussian blue analogs are great candidates of electrode materials for multivalent ion storage thanks to the intrinsic open pore structure enabling reversible intercalation of various ions. For example, Cui et al. demonstrated that reversible insertion of divalent ions (Mg^2+^, Ca^2+^, Sr^2+^, and Ba^2+^) was realized in nickel hexacyanoferrate, NiHCF, with specific capacities of 30–60 mAh g^−1^ in aqueous electrolyte and good recyclability (2000 cycles) without capacity fading.^[^ ^63^ ^]^ Their subsequent work showcased the insertion of a wide range of divalent and trivalent ions, such as Rb^+^, Pb^2+^, Al^3+^, and Y^3+^, was allowed in CuHCF with good capacity and stability.^[^ ^64^ ^]^

Zinc ion batteries (ZIBs) using PBA as cathode materials are the most intensively studied among these multivalent ion batteries. Trócoli and La Mantia reported an aqueous Zinc‐ion battery based on CuHCF in zinc sulfate solution electrolyte, which showed a voltage of 1.73 V and cyclability, rate capability, and specific energy (45.7 Wh kg^−1^ at 60 mA g^−1^ to 33.8 Wh kg^−1^ at 10 C) that was comparable to lithium‐ion organic batteries.^[^ ^65^ ^]^ Endres et al. demonstrated that a zinc battery with a combination of PBA (FeHCF) cathode and biocompatible electrolyte (bio‐ionic liquid‐water mixture), exhibited a discharge voltage plateau of ≈1.1 V with a specific capacity of about 120 mAh g^−1^ at 0.1 C.^[^ ^66^ ^]^

A high‐performance zinc ion battery was realized by activation of C‐coordinated low spin Fe in FeHCF cathode through high‐voltage scanning at 2.3 V.^[^ ^67^ ^]^ The Zn–FeHCF hybrid‐ion battery achieved a specific capacity of 74.6 mAh g^−1^ within 300 cycle, and exceptional cycling performance of 5000 and even 10 000 cycles with 82% and 73% capacity retention, respectively. Besides, 53.2% capacity of the rate capability was maintained at current density of 8 A g^−1^. TEM and ex situ XRD studies revealed that the reversible insertion/extraction of zinc and lithium ions were accompanied by the lattice distortion/recovery at discharge and charged state, respectively, which significantly enhanced the cycling performance.

Rechargeable Ca‐ion battery with PBA cathode (MnHCF) was developed by Ingram et al. in a nonaqueous electrolyte, with a specific capacity of 80 mAh g^−1^.^[^ ^68^ ^]^ Reversible intercalation of calcium ions was realized in the MnHCF cathode. Yao et al. demonstrated an aqueous Ca‐ion battery composed of polyimide anode and CuHCF cathode that exhibited a stable capacity (40 mAh g^−1^) and specific energy (54 Wh kg^−1^) at both high and low current rates, with an 88% capacity retention and an average 99% Coulombic efficiency (1000 cycles at 10 C).^[^ ^69^ ^]^

Mg‐ion batteries and Al‐ion batteries were also developed using Prussian blue analogs as cathode materials. A Mg‐ion battery with a CuHCF cathode in an aqueous electrolyte was reported by Okubo et al., which delivered a specific capacity of 50 mAh g^−1^ at a current rate of 0.1 A g^−1^.^[^ ^70^ ^]^ CuHCF cathode was also found capable of reversibly inserting/extracting Al^3+^ ions in an aqueous Al‐ion battery with a discharge capacity of 62.9 mAh g^−1^ at 50 mA g^−1^.^[^ ^71^ ^]^ Subsequently, Xia et al. reported an aqueous Mg‐ion battery with a NiHCF cathode and a polyamide anode, exhibiting a capacity of 33 mAh g^−1^ at the current density of 1 A g^−1^.^[^ ^72^ ^]^ A good cyclability was demonstrated by a 60% capacity retention after 5000 cycles.

PBA materials have been recognized as promising host materials for high‐performance rechargeable batteries. Other ion batteries using PBA as electrodes are also emerging such as proton batteries^[^ ^73^ ^]^ and ammonium batteries.^[^ ^74^ ^]^ However, problems still exist in this class of materials for practical applications, including low discharge capacity and poor cycling stability. To overcome these challenges, new synthetic methodology should be proposed to optimize the crystallinity and reduce the vacancies and water content, which will facilitate the insertion of cations.

### 2.4 Spin Crossover

Spin crossover (SCO) materials are a class of materials that shows dynamic switching between high‐spin (HS) and low‐spin (LS) states upon external stimuli, such as guest molecules, thermal treatment, pressure, and light.^[^ ^75^ ^]^ Spin crossover (SCO) phenomena have been observed in metal complexes containing transition metal ions with *d* ^4^–*d* ^7^ electronic configurations. Specifically, the nature of spin crossover behavior in a particular compound is governed by the ligand field splitting on the HS and LS states. In a suitable ligand field, SCO can be triggered thermally while it can also be induced by other perturbations like light and pressure.^[^ ^76^ ^]^ Therefore, their electronic, magnetic, optical, and mechanical properties can be precisely controlled by manipulating the spin transition, which sets a platform to investigate their applications in sensing and information storage.^[^ ^77^ ^]^

Extensive research efforts have been devoted to compounds based on cyanide, a strong field ligand that usually leads to a low spin state in C‐coordinated metal ions, which enables “ON‐OFF” spin transition between diamagnetic state (*S* = 0) and paramagnetic (*S* = 2) state in metal sites with *d* ^6^ configuration, such as Fe(II). Therefore, an important landmark of this journey is the investigation of Hofmann‐type coordination polymers, in which these two moieties can be integrated into a spin crossover system.^[^ ^78^ ^]^ To realize practical applications of switchable molecular materials, system showing abrupt spin crossover near room temperature with wide hysteresis is desired. Trends in the SCO research community have been emerging in chemical compositional modulations, multistable SCO systems, multiresponsive SCO systems, and fabrication of downsized SCO systems.

#### 2.4.1 Chemical Compositional Modulations

The spin transition is transmitted cooperatively throughout the crystal lattice. Strong SCO cooperativity is favored for hysteretic transition and well‐defined bistability due to the efficient communications among metal centers, which relies on the intermolecular interactions, including host‐host and host‐guest interactions. Therefore, numerous attempts were made for fine‐tuned SCO systems to fit in practical applications, by various strategies, such as tailoring the organic linkers and cyanometallate ligands, and controlling the inclusion/extraction of guest molecules.

##### Host Structure Modulations

SCO properties can be significantly tuned by chemical modulations on metal ions and linkers. Systematic investigation on the SCO properties in a series of 12 2D Hofmann‐type MOFs{Fe(3‐Xpy)_2_[M^II^(CN)_4_]} (M^II^: Ni, Pd, Pt; X: F, Cl, Br, I; py: pyridine) featuring square planar [M^II^(CN)_4_]^2−^ ligands, demonstrated the effect of chemical compositions on the spin transition behaviors.^[^ ^79^ ^]^ All the fluoro and chloro derivatives underwent cooperative spin crossover (SCO) with significant hysteretic behavior, whereas the bromo and iodo derivatives are paramagnetic. From the critical temperature of these compounds, it can be concluded that by moving the substitution group from F to Cl and the cyanometallates ligand from Pd and Pt to Ni, the LS state was destabilized and the cooperativity was weakened due to the different electronegativities and polarizabilities. Interestingly, compound ClPd, which crystallized in a different space group, can adopt an intermediate phase with mixed LS/HS spin states and display a two‐step SCO behavior. Such a phenomenon suggested that subtle coordination environment and symmetry distortion would also significantly affect the overall SCO behaviors.

The high tunability of SCO behavior was further showcased by employing solid solution as an efficient strategy to manipulate the spin transition and cooperativity. Gu et al. reported the incorporation of linkers with different functional groups (A: pyrazine, B: amino‐pyrazine, C: quinoxaline, D: 5,6,7,8‐tetrahydroquinoxaline) in a series of 3D Hofmann‐type MOFs based on [M^II^(CN)_4_]^2−^ ligands, Fe(L)[M^II^(CN)_4_] (M^II^: Pd, Pt).^[^ ^80^ ^]^ Distinct magnetic properties were shown in four isostructural compounds: only Fe(A)[Pt(CN)_4_] exhibited SCO at near room temperature with hysteresis loop while Fe(B)[Pt(CN)_4_], Fe(D)[Pt(CN)_4_] were in HS state and Fe(C)[Pt(CN)_4_] was in LS state. By tuning the portion of the pillar ligands in Fe(A)*_x_* (B)_1−_ *_x_* [Pt(CN)_4_] from *x* = 0 to 1, the critical temperature *T*_c_^↑^ and *T*_c_^↓^ were adjusted from 239 to 254 K and from 248 to 284 K, respectively. What's more, the hysteresis loop width was also widened from 9 to 30 K. This method was also applicable to Pd derivatives.

**Figure 10:**
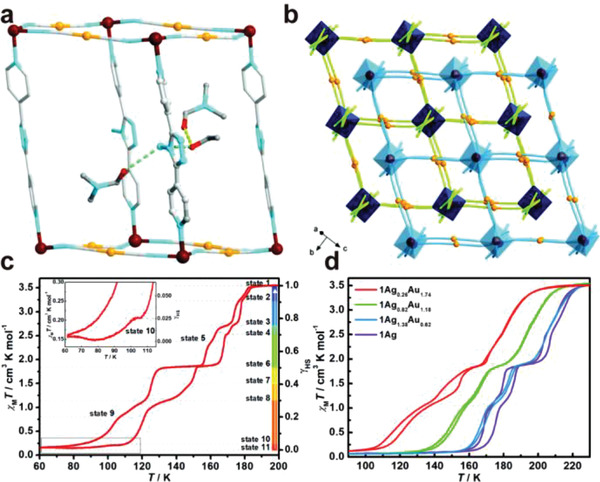
a) Crystal structure of [Fe(4‐abpt){Au(CN)_2_}_2_]·2DMF·EtOH (1Au) at 240 K. b) Doubly interpenetrated framework. Thermal dependence of *χ*_M_ *T* for c) 1Au and d) 1Ag_0.26_Au_1.74_, 1Ag_0.82_Au_1.18_, 1Ag_1.38_Au_0.62_ and 1Ag. Reproduced with permission.^[^ ^81^ ^]^ Copyright 2020, Royal Society of Chemistry.

The use of solid solution strategy for control of SCO behavior can also be realized by tuning the content of cyanometallate ligands. In the [Fe(4‐abpt){M^I^(CN)_2_}_2_]·2DMF·EtOH (4‐abpt = 4‐amino‐3,5‐bis(4‐pyridyl)‐1,2,4‐triazole) system, different ratios of the linear [M^I^(CN)_2_]^−^ (M = Ag, Au) ligands can be incorporated from pure Au to Ag, without changing the topology of the doubly interpenetrated framework^[^ ^81^ ^]^ (**Figure** **10**a,b). 1Au exhibited exceptional hysteretic asymmetric seven‐/eight‐step SCO behavior, which was changed to four‐step SCO when introduced with Ag (Figure 10c,d). More importantly, the critical temperature was further shifted higher by increasing the content of Ag in the framework from 1Ag_0.26_Au_1.76_ to 1Ag. The weaker ligand field and higher linearity in [Au(CN)_2_]^−^ facilitated the distortion of the coordination geometry of Fe and pillar ligand, which resulted in the change of SCO behavior.

Besides, modification of structural topology by incorporating different cyanometallate ligands is another efficient way to control SCO properties. Gural'skiy et al. reported the distinct SCO behaviors in a series of Hofmann‐type coordination polymers, {Fe^II^(Mepz)[Au^I^(CN)_2_]_2_} ([Au]) and {Fe^II^(Mepz)_2_[Ag^I^(CN)_2_]_2_} ([Ag]) (Mepz = 2‐methylpyrazine).^[^ ^82^ ^]^ The complex [Au] exhibited an incomplete stepped spin transition as a function of the temperature with *T*_SCO1_ = 170 K and *T*_SCO2_ = 308 K for the two subsequent steps. In contrast, the complex [Ag] attained the high‐spin state over the whole temperature range. It is concluded that the weak interlayer contacts (Ag−*π*, Me−*π*, and Ag−N) might be responsible for an unusual axial elongation of the FeN_6_ polyhedra. Real et al. discovered an in situ generated elongated [Ag_2_(CN)_3_]^−^ linker in a triply interpenetrated framework {Fe(2,6‐naphthy)[Ag(CN)_2_][Ag_2_(CN)_3_]} (2,6‐naphthy = 2,6‐naphthyridine) (1) compared to the prototypical doubly interpenetrated {Fe(2,6‐naphthy)[Au(CN)_2_]_2_}·0.5PhNO_2_ (2).^[^ ^83^ ^]^ The densely packed 1 exhibited a relatively abrupt two‐step SCO in the temperature interval 150–215 K, whereas 2, with nitrobenzene as guest molecules, featured an incomplete one‐step SCO behavior (*T*_1/2_ = 166 K) that extended over 150 K.

##### Host–Guest Interaction Modulations

Guest molecules have significant effect on manipulating the SCO properties through host‐guest interactions. In the 3D Hofmann‐type MOF Fe(pyrazine)_2_Pt(CN)_4_, different SCO behaviors were shown when loaded with three different ring aromatic guest molecules, furan, pyrrole, and thiophene, termed as 1·fur, 1·pyr and 1·thio, respectively.^[^ ^84^ ^]^ 1·fur exhibited complete and two‐step transition, while only 50% spin transition was shown in 1·pyr and 1·thio when cooling. Computational calculations revealed that high spin (HS) states were stabilized by the host–guest interactions with the bulkier compounds (pyrrole and thiophene). Neville et al. reported that the change of SCO behavior in a 2D [Fe(thtrz)_2_Pd(CN)_4_]·EtOH,H_2_O (1·EtOH, H_2_O, thtrz = *N*‐thiophenylidene‐4*H*‐1,2,4‐triazol‐4‐amine) upon guest removal.^[^ ^85^ ^]^ Both saturated phase 1·EtOH, H_2_O and 1·3H_2_O exhibited two‐step SCO behavior, whereas the intermediate plateau with HS^0.5^LS^0.5^ configuration was lost in dehydrated phase 1·H_2_O and 1, which showed higher critical temperature. It is concluded that the guest‐dependent “ON‐OFF” switch between different stepped SCO transitions is attributed to the elastic frustration and lattice flexibility modulated by the guest molecules.

In many cases, the removal of guest molecules results in the loss of host–guest interactions, negatively affecting the cooperativity. However, the spin state communication was demonstrated to be strengthened in a flexible 2D Hofmann‐type MOF with ligands featuring strong aromatic interaction sites.^[^ ^86^ ^]^ The dehydration facilitated the rearrangement of ligand orientations and close host‐host contacts in the crystal lattice, which realized optimal cooperativity and a combination of room temperature SCO and wide hysteresis.

#### 2.4.2 Multistable SCO Systems

Design and synthesis of materials that exhibit multiple stepped SCO transitions with well‐defined hysteresis are promising for new “smart” electronic switching and data storage. Kepert et al. reported unusual and complex four stepped hysteretic SCO behavior in a doubly interpenetrated 3D Hofmann‐type MOF with linear [Au^I^(CN)_2_]^2−^ building blocks, [Fe^II^(bipydz)(Au^I^(CN)_2_)_2_]·4(EtOH) (**1** ^AS^; bipydz = 3,6‐bis(4‐pyridyl)‐1,2‐diazine).^[^ ^87^ ^]^ Variable‐temperature magnetic susceptibility measurements revealed a spin transition from fully HS, HS_0.67_LS_0.33_, HS_0.5_LS_0.5,_ HS_0.67_LS_0.33_, HS_0.33_LS_0.67_ to fully LS states when cooling. X‐ray diffraction experiments confirmed the structural transformations during spin transitions, which originated from the generation of inequivalent Fe^II^ sites, giving new lattice symmetry and periodicity in different integral HS fractions. It was found that host–host and host–guest interactions in [Fe^II^(bipydz)(Au^I^(CN)_2_)_2_]·4(EtOH) were crucial to the stabilization of the intermediate spin states.

Another type of hysteretic four‐step SCO behavior was demonstrated by Tong et al. in a 3D Hofmann‐type MOF [Fe(4‐abpt){Ag(CN)_2_}_2_]·2DMF·EtOH (4‐abpt = 4‐amino‐3,5‐bis(4‐pyridyl)‐1,2,4‐triazole), showing the sequence of LS↔HS_0.25_LS_0.75_↔HS_0.5_LS_0.5_↔HS_0.75_LS_0.25_↔HS.^[^ ^88^ ^]^ Five distinct spin state patterns that correspond to different spin‐states were structurally identified, in which the four different Fe^II^ sites in 1‐iv with HS_0.25_LS_0.75_ configuration exhibited the unprecedented —LS—LS—LS—HS— 3D spin‐state ordering in all stripes. More importantly, the stepped SCO behavior in this system can be reversibly adjusted to two‐, and one‐stepped SCO by guest exchange.

**Figure 11:**
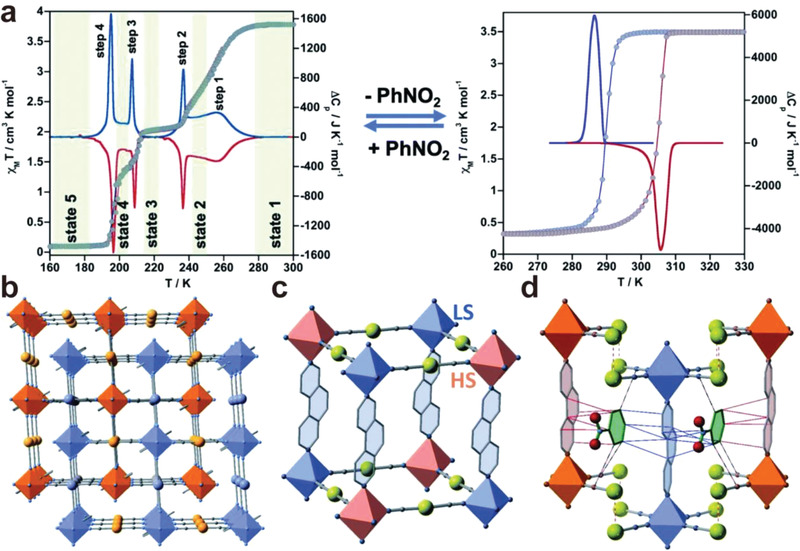
a) Thermal dependence of *χ*_M_ *T* and calorimetric properties for {Fe^II^(3,8‐phen)[Au(CN)_2_]_2_}·*x*PhNO_2_ (1·PhNO_2_)(left) and 1 (right). b) Two‐fold interpenetrated networks. c) Ordering of LS (blue) and HS (pink) metal centers in the state 3 with HS_0.5_LS_0.5_ configuration. d) Host–guest interactions highlighted in 1·PhNO_2_. Reproduced with permission.^[^ ^89^ ^]^ Copyright 2021, Royal Society of Chemistry.

Other than the protic solvent molecules, aromatic guest molecules, such as benzene derivatives were found to promote multi‐stepped SCO. Real et al. reported a reversible on–off switching of elastic frustration in a 3D {Fe^II^(3,8‐phen)[Au(CN)_2_]_2_}·*x*PhNO_2_ (1·*x*PhNO_2_) (3,8phen = 3,8‐phenanthroline, PhNO_2_ = nitrobenzene) enabled by the insertion/extraction of PhNO_2_.^[^ ^89^ ^]^ With the inclusion of PhNO_2_ molecules, spin transition with a sequence of LS↔HS_0.33_LS_0.67_↔HS_0.5_LS_0.5_↔HS_0.67_LS_0.33_↔HS was exhibited, while the guest‐free derivative **1** showed hysteretic single‐step SCO behavior at room temperature (**Figure** **11**a). The molecular recognition of PhNO_2_ via host‐guest *π*–*π* interactions enables “molecular wedge” effect between the interpenetrated frameworks, which facilitated elastic frustration and multistep behavior (Figure 11b–d). These works have provided a pathway to explore the long‐range ordering of Fe^II^ sites and the stabilization of guest molecules for multistep spin‐crossover systems.

#### 2.4.3 Multiresponsive SCO Systems

Other than thermal treatment, the spin transition can be induced by other stimuli, such as external pressure and light irradiation. Switchable magnetic property enabled by tunable transition between low‐spin (LS) and high‐spin (HS) states via various stimuli is important for the pursuit of information storage devices.

Multiresponsive SCO behaviors have been demonstrated in SCO systems featuring different cyanometallate ligands. For example, Real et al. reported a 2D Hofmann‐type MOF using [Pd(CN)_4_]^2−^ ligands, [Fe(3‐Clpy)_2_Pd(CN)_4_] (3‐Clpy = 3‐chloropyridine), which showed a thermal‐induced two‐step spin crossover (*T*_c1_^↓^ = 159.6 K, *T*_c1_^↑^ = 164.5 K, *T*_c2_^↓^ = 141.4 K and *T*_c2_^↑^ = 148.4 K).^[^ ^90^ ^]^ When irradiated with green light (514 nm) at low temperature (10 K), around 60 % of the low‐spin (LS) iron(II) centers in this complex were excited to high‐spin state (LIESST effect). The LIESST relaxation kinetics studies revealed that the cooperativity was resulted from the elastic interactions induced by the metal–ligand bond lengths change during spin transitions. The spin transition of this compound can also be triggered by external pressure. At 300 K, **1** underwent a cooperative spin‐crossover transition at around 0.6 GPa.

**Figure 12:**
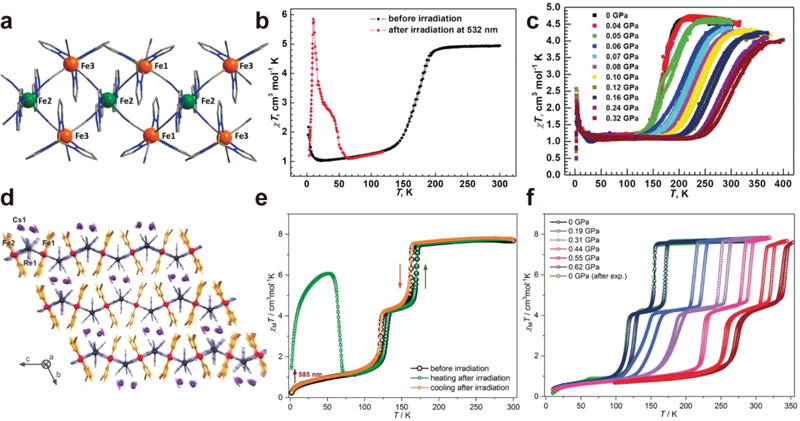
a) Crystal structure of {[(Tp)Fe^III^(CN)_3_]_2_Fe^II^(azp)·4H_2_O. Thermal dependence of *χ*_M_ *T* before and after irradiation b) and under different external pressure c). Reproduced with permission.^[^ ^91^ ^]^ Copyright 2018, Wiley‐VCH. d) Crystal structure of Cs{[Fe^II^(3‐CNpy)_2_][Re^V^(CN)_8_]}·H_2_O. Thermal dependence of *χ*_M_ *T* before and after irradiation e) and under different external pressure f). Reproduced with permission.^[^ ^92^ ^]^ Copyright 2020, Wiley‐VCH.

Liu et al. reported the multi‐responsive SCO behaviors in a cyanide‐bridged {Fe^III^Fe^II^} chain, {[(Tp)Fe^III^(CN)_3_]_2_Fe^II^(azp)·4H_2_O (azp = 4,4'‐azopyridine), in which the Fe^II^ sites were linked by azp ligands and negative charged magnetic building blocks [(Tp)Fe(CN)_3_]^−^ (Tp = hydrotris(pyrazolyl)borate)^[^ ^91^ ^]^ (**Figure** **12**a). Before irradiation, this complex showed spin transition (*T*_1/2_ = 171 K) from 1.05 cm^3^ mol^−1^ K at 20 K to 4.95 cm^3^ mol^−1^ K at 200 K (Figure 12b). Light irradiation at 532 nm under cryogenic temperature triggered spin transition from low‐spin to high‐spin states in Fe^II^ sites, which led a sharp maximum of 5.85 cm^3^ mol^−1^ K at 10 K. The photoinduced magnetization was recovered to the initial value at elevated temperature of 66 K. Furthermore, the response to external pressure of this complex was confirmed by pressure‐dependent SCO behavior studies, which revealed that the critical temperature can be tuned from 171 K at ambient pressure to 301 K at 0.32 GPa with well‐defined hysteresis loop (Figure 12c).

What's more, octacyanometallate ions are also demonstrated to be a great candidate as linkers for construction of multi‐responsive SCO systems. Sieklucka et al. reported the Cs{[Fe^II^(3‐CNpy)_2_][Re^V^(CN)_8_]}·H_2_O (3‐CNpy = 3‐cyanopyridine) network, in which 2D cyano‐bridged Fe‐Re square grid layers were interconnected by Cs^+^ ions^[^ ^92^ ^]^ (Figure 12d). This complex exhibited thermally induced two‐stepped spin crossover due to the presence of two different Fe sites which led to an intermediate phase with LS_0.5_HS_0.5_ configuration. This compound also exhibited LIESST effect with high photoconversion of over 80% when irradiated with visible light (585 nm) (Figure 12e). The thermally induced two‐stepped SCO behavior was retained at elevated pressure even up to 0.62 GPa, while the transition temperature was moved to higher temperature as a result of the stabilization of LS state under high pressure (Figure 12f).

Photoswitchable SCO systems have attracted more and more interest due to its applications in various properties, such as the design and synthesis of photomagnet,^[^ ^93^ ^]^ chiral optical switching magnet.^[^ ^94^ ^]^ Interestingly, the light‐induced magnetic response can be enforced by applying external mechanical pressure in a nonphotomagnet.^[^ ^95^ ^]^ Besides, new manipulation of light‐ or temperature‐assisted spin state annealing (LASSA and TASSA) was demonstrated effective in discovering hidden spin states and new multistability in SCO system.^[^ ^96^ ^]^ Therefore, by modulating external stimuli, increasing number of new functions in materials with multi‐stable and multiresponsive features is expected in the future.

#### 2.4.4 Downsized SCO Systems

Most studies on SCO complexes are focused on the bulk samples. However, materials for solid‐state sensor, optical devices, and memory applications, will be downsized as nanoparticles or fabricated as thin films. When downsized, the magnetic properties and cooperativity of an SCO material are drastically affected since the surface chemistry on the interface of nanoparticle indeed plays important role in changing the collective behavior of the SCO centers.^[^ ^97^ ^]^ For example, the spin transition of downsized nanoparticle Fe(pz)[Pt(CN)_4_]} (7, 14 nm), which exhibited SCO behavior with large hysteresis in the bulk state, was shifted to lower temperature with unclear hysteresis.^[^ ^98^ ^]^ In most cases, the reduced dimensions led to a loss of well‐defined SCO properties.

The structure–SCO property relations have been investigated in several downsized systems. By using an optimized layer‐by‐layer strategy, the fabrication of a series of 2D [Fe(py)_2_{Pt(CN)_4_}] (py = pyridine) thin film with thickness ranging from 40 to 0.7 nm (30 to 1 layer) was successfully realized by Martí‐Gastaldo et al.^[^ ^99^ ^]^ The dependence of the SCO behaviors on the downsizing effect in the thin films was investigated AFM and synchrotron X‐ray absorption spectroscopy (XAS) due to the limitation of magnetic measurement for samples in nanoscale, which revealed that the SCO transition was retained down to a critical value of 12 nm, while the spin transition and cooperativity were lost when reduced to smaller sizes. When the number of growth cycles is below the 15‐layer limit, the fraction of HS Fe^II^ center abruptly increased from 20% in 15‐cycle film to 67% in the 1‐cycle film. However, the topography of the thin film obtained from AFM clearly showed that the lateral particle size remains almost constant (26 ± 6 nm), which indicated that the changes in the SCO transition were correlated to the variations in the degree of coalescence in the thin films (C). When the crystallites on the film fully coalesced and formed homogenous film with densely packed particles, the HS fractions in the thin film became similar to that of bulk state. Therefore, other than thickness, the nanostructure of ultrathin film and underlying interparticle interactions play a crucial role in affecting the cooperativity and SCO behavior.

Attempts have been made to realize the control of SCO behavior in downsized form. Real et al. reported the chemical modulations of ligand composition in the [Fe(py)_2_{Pt(CN)_4_}] system from pyrazine (py), pyrimidine (pym) to isoquinoline (isoq).^[^ ^101^ ^]^ Although cooperative and complete SCO properties were retained in FePyPt films (60 cycles), a different growth mechanism of FePymPt and FeIsoqPt film had led to an increased fraction of HS Fe^II^ sites. Therefore, a complete loss of cooperativity for FeIsoqPt and an asymmetric and incomplete hysteresis loop for FePymPt was observed.

**Figure 13:**
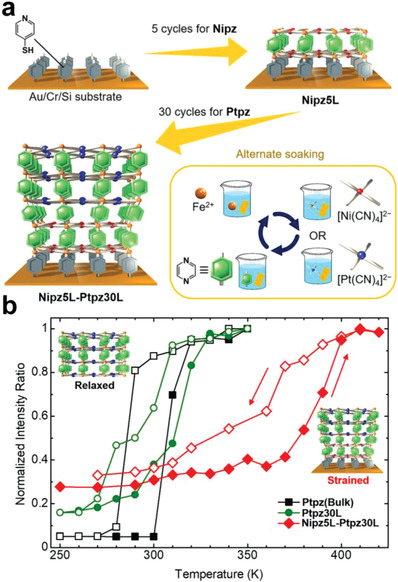
a) The fabrication of a heterostructured thin film (Nipz5L‐Ptpz30L) via LbL approach. b) Temperature dependence of the normalized Raman intensity ratio. Reproduced with permission.^[^ ^100^ ^]^ Copyright 2021, American Chemical Society.

Very recently, a new strategy of strain‐controlled spin transition was proposed by Kitagawa et al. to manipulate the SCO behavior of MOF thin film.^[^ ^100^ ^]^ A heterostructured thin‐film Nipz5L‐Ptpz30L, (pz = pyrazine) composed of 5 layers of {Fe(pz)[Ni(CN)_4_]} (Nipz) as a bottom buffer layer and 30 layers of {Fe(pz)[Pt(CN)_4_]} (Ptpz) was constructed to introduce compressive strain to the Ptpz layer due to the larger lattice in Ptpz (**Figure** **13**a). The spin transition temperature of the heterostructured film was increased from 300 to 380 K (Figure 13b). The compressive strain to Ptpz layers induced a shorter distance between Fe^II^ and CN ligands and weakened steric repulsion of pyrazine and CN ligands, which facilitated a strong ligand field on Fe^II^ sites and stabilized LS states entropically. This mechanism demonstrated the feasibility of controlling the SCO behavior of MOF films by fabricating heterostructured thin film.

To summarize, although significant advances have been achieved in SCO nanomaterials in the past decade, challenges still remained in the control of thin‐film fabrications, characterizations of crystal structures, electronic configurations and magnetic properties, and the fundamental understanding of the downsizing effect. The potential of SCO nanomaterials should be fully explored in the future.

## 3 Conclusions

From a modern perspective, the chemistry of metal–ligand coordination bond had revolutionized the development of crystalline periodic solid‐state material, as exemplified by porous coordination polymers or metal‐organic frameworks. However, in comparison with the exponentially growing number of new inorganic‐organic hybrid materials, cyanometallates, a diverse class of old molecular building blocks, have been somewhat forgotten in the past decade. Indeed, the inexpensive cyanide ligands, its unique chemistry and versatile cyanometallates possessing different symmetries and coordination numbers, can serve as a great platform for the exploration of novel multifunctional materials.

Intensive research efforts have been devoted to pore and function engineering for various specific applications. In this regard, we have outlined the progress of cyanide‐based materials in several representative applications including gas adsorptions and separations, electrocatalysis, energy storage, and SCO materials. Specifically, pore engineering strategy was proven highly effective in realizing guest molecule induced gate‐opening effect and optimizing the separating performance for harmful gases removal, hydrocarbon purifications. Substantial progress had been made in the applications of energy conversion and storage using cyanide‐based materials promoted by the embedded active sites and redox nature. Multistable and multiresponsive SCO materials are continuously emerging for new information storage materials. To target the next‐generation top‐performing multifunctional materials, the exploration of fundamental design principles and underlying structure‐property relationships is of great importance. Thereby, the highly tunable feature of cyanometallates with respect to chemical compositions, functionalities and coordination geometries provides an extraordinary opportunity to conduct in‐depth studies and to solve the puzzling of optimal performance. In contrast to other functional materials, the metal cyanide‐based MOFs can simultaneously combine compact pore structure and efficient electronic/magnetic coupling thanks to the short cyanide linker, which renders them as versatile promising materials especially for those with multi‐functionality. With continuous research efforts devoted in this field, more and more new functionalities with top performance and novel extended networks are anticipated by using these old cyanometallates building blocks.

In spite of the great research progress, several challenges should be addressed as research on multifunctional cyanide‐based materials go wider and deeper. i) The mechanism of nanomaterial synthesis and morphology control is still lacking. The nucleation and fabrication of thin films or nanoparticles are yet to be fully investigated in many applications, such as SCO properties. The performance of rechargeable batteries using cyanide‐based materials is hindered by crystal imperfections and vacancies. Morphology regulation of electrocatalyst for higher active site number and energy conversion efficiency is still underdeveloped. ii) New characterization technologies, especially in situ characterizations, are crucial to reveal the nanostructures and dynamic structural evolutions. For example, precise characterizations of the diverse distribution of vacancies and the mixed valences/spin states from transition metal ions within the crystals are still highly challenging. Advanced technologies such as Raman spectroscopy, X‐ray absorption spectroscopy and HRTEM should be utilized for new insights into the underlying mechanisms. iii) Integration of cyanide‐based materials with composites like carbon materials, will help overcome its intrinsic limit, such as the poor conductivity for electrochemical processes. To sum up, new dimensions of research toward rational design of optimal separation agents, switchable materials, and hybrid composites based on cyanide‐bridged materials are currently emerging and have attracted more and more attention. The exploration of multifunctional materials with cyanide building blocks is promising for practical applications, and even commercial purposes.

## Conflict of Interest

The authors declare no conflict of interest.
